# Conformal Self-Assembly of Nanospheres for Light-Enhanced Airtightness Monitoring and Room-Temperature Gas Sensing

**DOI:** 10.3390/nano11071829

**Published:** 2021-07-14

**Authors:** Qirui Liu, Yinlong Tan, Renyan Zhang, Yan Kang, Ganying Zeng, Xiaoming Zhao, Tian Jiang

**Affiliations:** 1 College of Advanced Interdisciplinary Studies, National University of Defense Technology, Changsha 410073, China; lqrkili@163.com (Q.L.); ryancms@sina.cn (R.Z.); 13321112309@163.com (Y.K.); zengganying@nudt.edu.cn (G.Z.); 2 Beijing Institute for Advanced Study, National University of Defense Technology, Beijing 100000, China; 3 State Key Laboratory of High Performance Computing, College of Computer Science and Technology, National University of Defense Technology, Changsha 410073, China; zxm_0911@126.com

**Keywords:** nanospheres, tungsten disulfide, conformal self-assembly, light-matter interaction, gas sensor, airtightness monitor

## Abstract

The fabrication of conformal nanostructures on microarchitectures is of great significance for diverse applications. Here a facile and universal method was developed for conformal self-assembly of nanospheres on various substrates including convex bumps and concave holes. Hydrophobic microarchitectures could be transferred into superhydrophilic ones using plasma treatment due to the formation of numerous hydroxyl groups. Because of superhydrophilicity, the nanosphere suspension spread on the microarchitectures quickly and conformal self-assembly of nanospheres can be realized. Besides, the feature size of the conformal nanospheres on the substrates could be further regulated by plasma treatment. After transferring two-dimensional tungsten disulfide sheets onto the conformal nanospheres, the periodic nanosphere array was demonstrated to be able to enhance the light harvesting of WS_2_. Based on this, a light-enhanced room-temperature gas sensor with a fast recovery speed (<35 s) and low detecting limit (500 ppb) was achieved. Moreover, the WS_2_-covered nanospheres on the microarchitectures were very sensitive to the changes in air pressure due to the formation of suspended sheets on the convex bumps and concave holes. A sensitive photoelectronic pressure sensor that was capable of detecting the airtightness of vacuum devices was developed using the WS_2_-decorated hierarchical architectures. This work provides a simple method for the fabrication of conformal nanospheres on arbitrary substrates, which is promising for three-dimensional microfabrication of multifunctional hierarchical microarchitectures for diverse applications, such as biomimetic compound eyes, smart wetting surfaces and photonic crystals.

## 1. Introduction

The utilization of conformal nanostructures formed on microarchitectures is of great significance for diverse applications, such as wetting tunability [1,2], flexible electronics [3,4], photodetectors [5], biomimetic photonics [6] and green energy [7]. Although micro/nanostructure fabrication via broad-range techniques of photolithography [8], laser etching [9], three-dimensional (3D) printing [10], thermal oxidation [11] and chemical deposition [12] has been highly developed, a method for validly constructing conformal nanostructures on complex microarchitectures remains elusive, especially for large-area fabrication of periodic nanostructures on convex and concave surfaces. With the merit of surface wrinkling, mechanical-driven assembly of wrinkling patterns has been demonstrated as an efficient method to fabricate nanostructures on curved substrates [13,14]. Tan et al. [15] obtained hierarchical wrinkled papillae arrays on spherical substrates via 3D shrinking-induced wrinkling of nonuniform graphene oxide films. Li et al. [14] fabricated multiscale microarchitecture arrays through photoinduced wrinkling of 3D microstructures. However, it is difficult to control the orientation and the feature size of wrinkling patterns on microarchitectures. Recently, the self-assembly of nanostructures (e.g., nanospheres, nanorods and nanowires) on microarchitectures has attracted much attention due to simple operation and high efficiency [16,17,18,19]. As a low-cost commercial nanomodule with tunable diameter, polystyrene (PS) nanospheres have been widely used in the fabrication of photonic crystals and sacrificial templates for antireflective microstructures [20], low-temperature gas sensors [21], photodetectors [22] and electromagnetic absorption [23]. However, most previous studies mainly focused on hexagonal stacking of PS nanospheres on flat substrates, while few studies concentrated on the assembly of nanospheres on curved microarchitectures, which may limit their applications in the fabrication of biomimetic compound eyes [24,25] and micro/nanorobots [26]. Therefore, it is crucial to develop a highly efficient assembly method for the formation of conformal nanostructures on various curved microarchitectures, especially for convex and concave surfaces.

Because of versatile functions and low cost, the fabrication of high-performance room-temperature gas sensors has become more and more important for environmental protection, gas leak detection and sustainable development [27,28,29]. Through the synthesis and design of micro/nanostructures, such as one-dimensional tubes [30], two-dimensional (2D) sheets [31] and 3D hierarchical architectures [32], many efforts have been put into improving the recoverability, stability and especially sensitivity and selectivity of the gas sensor [33,34,35,36]. For example, Yin et al. [37,38,39,40] prepared a series of gas sensors based on metal oxide nanoparticles and nanocomposites, and the sensors exhibited high sensitivity and selectivity in the detection of low-concentration H_2_ and CO gas. Due to tunable bandgap and high surface-to-volume ratio, 2D materials have been demonstrated as promising candidates for room-temperature gas sensing [41,42,43,44,45], also showing high potential in photoactivation recently [46,47,48,49]. As a member of the transition metal dichalcogenide (TMDC) family, tungsten disulfide (WS_2_) is applicable for the fabrication of light-enhanced room-temperature gas sensors due to broadband spectral response and fast relaxation interval [50,51,52,53,54]. Polyakov et al. [51] fabricated Au-decorated WS_2_ nanotubes for light-enhanced room-temperature detection of low-concentration NO_2_ gas using periodical 530 nm illumination. Huo et al. [52] constructed WS_2_-based field-effect transistors that can respond to both oxidizing gas and reducing gas at room temperature. Although the performance of WS_2_-based gas sensors can be improved using light illumination, the room-temperature gas sensor still suffers from a long recovery time and poor stability. The combination of hierarchical microarchitectures with the enhanced active area and photoresponsive 2D materials may shine some light on addressing this problem.

Herein, a facile, highly efficient and universal self-assembly method for the fabrication of conformal nanospheres on curved substrates including convex bumps and concave holes is proposed. The formation of nanosphere-supported WS_2_ sheets can be achieved by immersing the nanosphere-covered substrate into the WS_2_ suspension. Similar to the hierarchical papillae on the rose petals, the conformal nanosphere arrays were demonstrated to be able to enhance the interaction between the light and WS_2_ sheets. Besides, the WS_2_ sheets suspended over the nanospheres are very sensitive to the air pressure since the contact between the WS_2_ sheets and the nanospheres can be regulated by varying the pressure. These advantages make the nanosphere-supported WS_2_ sheets promising candidates for the fabrication of light-enhanced room-temperature gas sensors and sensitive pressure sensors. Impressively, a light-enhanced gas sensor with a fast recovery speed (<35 s) and a low detection limit (500 ppb NO_2_) is realized under room temperature based on the WS_2_-decorated nanospheres. In addition, an optoelectronic pressure sensor that is capable of monitoring the airtightness of vacuum devices is also fabricated by suspending the WS_2_ sheets over the nanosphere-covered microarchitectures. The pressure sensor exhibits a high sensitivity of 22.07 kPa^−1^ within the pressure range of 30 to 140 Pa. Particularly, the sensitivity of the light-enhanced pressure sensor increases dramatically with the reduction in air pressure from 101 to 0.03 kPa, enabling the pressure sensor to monitor the airtightness of vacuum devices. The fabrication method for suspended 2D WS_2_ sheets over 3D conformal nanospheres provides a new strategy for high-performance gas sensors and pressure detectors.

## 2. Materials and Methods

### 2.1. Polystyrene Nanospheres

The PS nanosphere suspension was purchased from Hangzhou Nano-Mall Technology Co., Ltd. (Hangzhou, China). The weight percentage of the PS nanospheres of the suspension is 2.5%, which can be diluted by the addition of deionized water. The average diameter of the nanospheres is 200 nm.

### 2.2. Flat and Curved Substrates

Smooth Pt electrodes and SiO_2_ substrates were selected as the substrates to investigate the assembly of PS nanospheres on flat metal and inorganic surfaces; they were provided by Beijing Sino Aggtech Co., Ltd. (Beijing, China). Bumpy Au electrodes and porous Polyethylene terephthalate (PET) substrates were selected as the curved substrates to study the assembly of PS nanospheres on convex and concave substrates, respectively.

### 2.3. Plasma Treatment of the Substrates

Before plasma treatment, the substrates were immersed in ethanol for 10 min and then dropped into an ultrasonic cleaner for 10 min. After that, the substrates were put into the chamber of plasma generator (PDC-36G) produced by Hefei Kejing Materials Technology Co., Ltd. (Hefei, China). The power was fixed at 18 W and the output frequency was 13.56 MHz.

### 2.4. Conformal Self-Assembly of the PS Nanospheres

After ultrasonic cleaning and plasma treatment, the hydrophobic substrates became superhydrophilic, which renders the suspension of PS nanospheres easy to spread around the surface, forming a uniform conformal coating of nanospheres. The substrates were immersed into the suspension of PS nanospheres for 5 min and then lifted out. Conformal nanosphere coatings can be obtained on various flat and curved substrates after solvent evaporation.

### 2.5. Fabrication of the WS_2_-Decorated Gas Sensor

Bumpy Au interdigital electrodes deposited on flexible porous PET substrates were used as the electrodes. Then, the PS nanospheres were conformally coated on the plasma-treated Au electrodes and PET substrates, forming hierarchical nanostructured surfaces. After that, the conformal nanosphere-coated surface was sputtered with a thin Au coating to improve the conductive network. Then, a plasma treatment of the Au-coated surfaces was carried out to enhance the adhesion between WS_2_ sheets and the nanostructured surfaces. Finally, WS_2_ suspension with a concentration of 1 mg/mL was directly dropped onto the conformal nanosphere-coated surfaces, leading to the formation of WS_2_-decorated nanosphere arrays with enhanced light-harvesting capability.

### 2.6. Characterization

Field emission scanning electron microscope MIRA 3 (TESCAN, Brno, Czech Republic) was used to characterize the surface morphology of various samples. Before characterization, the samples were sputtered with thin Au nanofilms to enhance their conductivity. The element analysis of WS_2_ sheets was carried out via the X-MAX20 (Oxford, UK) detector.

### 2.7. Performance of the Gas Sensor

The performance of the gas sensor was characterized with CGS-MT, an optoelectronics gas sensor test platform produced by Beijing Sino Aggtech Co., Ltd. (Beijing, China). A gas chamber with an optically polished quartz window was used to investigate the effect of light illumination on the performance of the gas sensor. The standard gas including NO_2_, CH_2_O, CO and NH_3_ was purchased from Shanghai Weichuang Standard Gas Analytical Technology Co., Ltd. (Shanghai, China). The test gas was calibrated according to the standard of GB/T5274-1-2018 (Gas analysis—Preparation of calibration gas mixtures—Part 1: Gravimetric method for Class I mixtures). The gravimetric method is an internationally recognized calibration method. The purchased standard gas was prepared by the gravimetric method. The test gas with various concentrations can be obtained by varying the concentration of the purchased standard gas using compressed dry air as carrier gas. Two mass flow controllers were used to mix different gases.

### 2.8. Performance of the Pressure Sensor

To characterize the performance of the pressure sensor, a vacuum pump (RV8) produced by Edwards was used to reduce the pressure of the chamber, and a vacuum gauge (ZJ-52T/kf10-16) produced by Chengdu Ruibao Electronic Technology Co., Ltd. (Chengdu, China) was used to measure the pressure of the test chamber. A semiconductor laser was used to produce high-quality light illumination of 532 nm wavelength.

## 3. Results and Discussion

### 3.1. Conformal Assembly of Nanospheres on Flat and Curved Substrates

**Figure 1:**
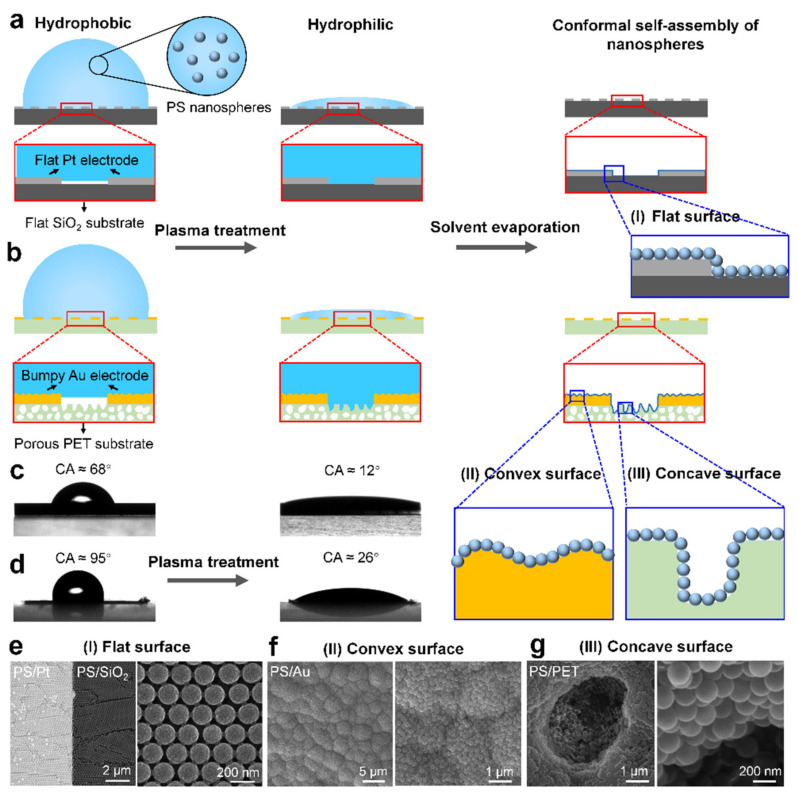
Conformal self-assembly of nanospheres on flat and curved substrates. (**a**,**b**) Schematics illustrate the fabrication process for conformal nanospheres on (**a**) flat and (**b**) curved substrates. (**c**,**d**) Improving the hydrophilicity of the substrates by plasma treatment: (**c**) Pt electrodes on SiO_2_ substrates; (**d**) Au electrodes on polyethylene terephthalate (PET) substrate. (**e**–**g**) SEM images show the conformal nanospheres on (**e**) flat Pt and SiO_2_ substrate, (**f**) convex Au substrate and (**g**) concave PET substrate.

Figure 1a,b depicts the assembly process of conformal nanospheres on flat and curved substrates. Firstly, plasma treatment was used to enhance the hydrophilicity of the substrates, and the substrates became superhydrophilic due to the introduction of numerous hydrophilic hydroxyl groups (Figure 1c,d). For example, the contact angle of the hydrophobic Au-decorated PET substrate decreased from 95° to 26° after plasma treatment (Figure 1d). Then, the plasma-treated substrates were immersed into the suspension of PS nanospheres with an average diameter of 200 nm. Due to the superhydrophilicity of the substrates, the suspension containing nanospheres quickly spread onto the surfaces and penetrated the concave holes, forming uniform liquid films on the substrates. After solvent evaporation, conformal self-assembly of nanospheres can be realized on both flat and curved substrates. Importantly, the developed method for the self-assembly of conformal nanostructures on microarchitectures is suitable for various substrates that have different shapes and surface chemicals, such as flat metal and inorganic surfaces (Figure 1e), convex metal surfaces (Figure 1f) and concave polymer holes (Figure 1g). The plasma-enhanced adhesion between the substrates and the nanospheres not only prevents the nanospheres from slipping off the convex bumps but also promotes the self-assembly of nanospheres in the spatial-confinement holes.

**Figure 2:**
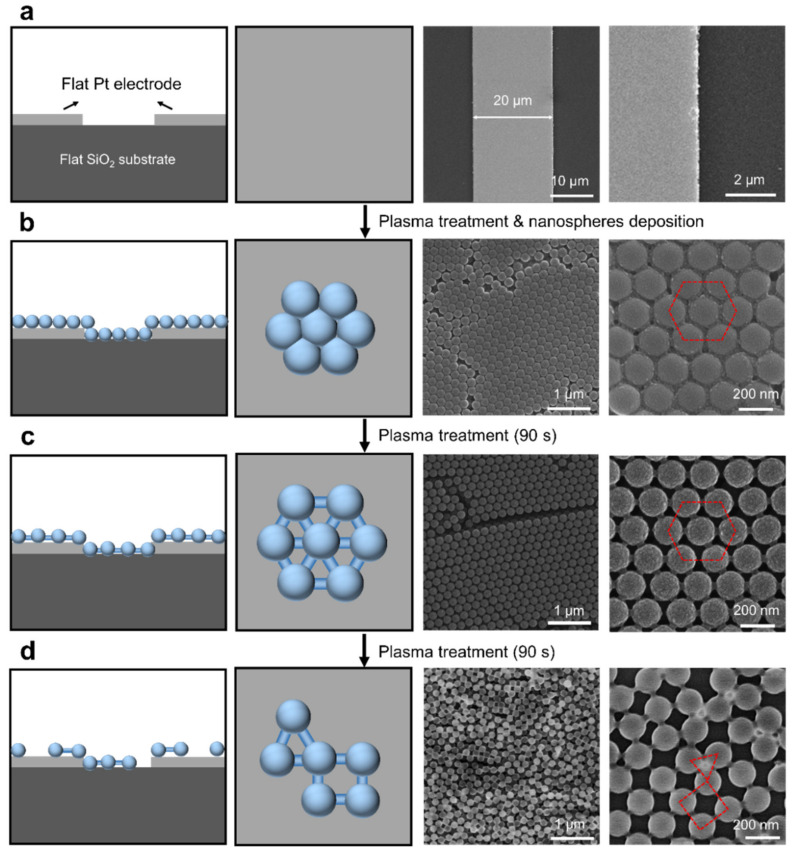
Controlling the feature size and the arrangement of the nanospheres on flat substrates via plasma treatment. (**a**–**d**) Schematics and SEM images show the morphology of (**a**) the flat Pt and SiO_2_ substrate, as well as the morphology evolution of the self-assembled nanospheres on the substrates with the variation of the plasma treatment time: (**b**) 0 s, (**c**) 90 s and (**d**) 180 s.

The morphology evolution of the plasma-treated nanospheres on flat substrates was then characterized to see the effect of plasma treatment on the feature size and the arrangement of the self-assembled nanospheres (Figure 2). The SEM images in Figure 2a show that the surfaces of Pt and SiO_2_ substrates are flat. When the nanosphere suspension was spread onto the substrates, the nanospheres self-assembled into close-packed hexagonal arrays (Figure 2b). After a plasma treatment of 90 s, the close-packed nanospheres transferred into interconnected hexagonal skeletons consisting of balls and sticks (Figure 2c). Interestingly, the obtained ball–stick skeleton is very similar to the honeycomb molecular skeleton of graphene. With a further increase in the plasma treatment time, much more plasma etching was concentrated on the nanospheres, and the interconnected hexagonal skeletons evolved into randomly distributed nanospheres (Figure 2d). Besides, the diameter of the nanospheres decreased from 200 to 160 nm with the increase in the plasma treatment time from 0 to 180 s (Figure S1). The developed method for the self-assembly of nanospheres into ordered interconnected hexagonal skeletons provides a simple approach for constructing artificial molecular models for metastructures.

**Figure 3:**
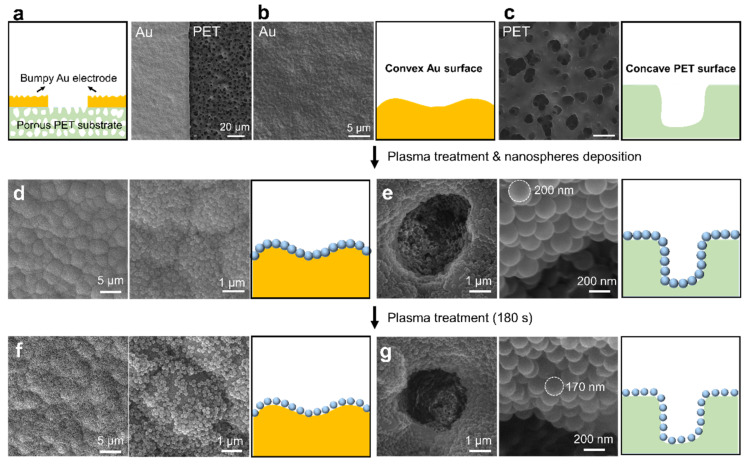
Conformal self-assembly of nanospheres on convex and concave substrates. (**a**–**c**) Schematics and SEM images of the substrates: (**a**) bumpy Au electrodes on porous PET substrate; (**b**) the undulating Au substrate; (**c**) the concave PET substrate. (**d**–**g**) SEM images and the schematics show the morphology of the nanosphere assembly on curved substrates (**d**,**e**) before and (**f**,**g**) after plasma treatment.

Although the assembly of nanospheres on flat substrates has been investigated intensively [55,56], the realization of a conformal assembly of nanospheres on convex bumps and concave holes is still challenging. With the use of the plasma treatment, the conformal assembly of nanospheres on curved substrates was next studied. As shown in Figure 3a–c, the undulating Au electrodes and the porous PET substrates were selected as the convex and concave surfaces to demonstrate the conformal assembly of nanospheres on curved substrates. As mentioned above, the substrates became superhydrophilic after plasma treatment. This makes it possible to realize the conformal assembly of nanospheres on bumps and holes. The nanosphere suspension spread quickly on the superhydrophilic substrates and the nanospheres were close-packed onto the substrates after solvent evaporation (Figure S2). It can be seen from the SEM images in Figure 3d,e that the nanospheres conformally covered on the Au microbumps and the PET microholes. Impressively, the nanospheres were close-packed on the inner surface of the microholes instead of filling up them, so the multilayer stacking of the nanospheres was avoided. Similarly, the diameter of the self-assembled nanospheres could be further reduced by additional plasma treatment. Particularly, the diameter of the nanospheres decreased from 200 to 170 nm after 180 s plasma treatment, as illustrated in Figure 3f,g (Figure S3). It is worth mentioning that the diameter of the nanospheres was reduced to 160 nm on a flat SiO_2_ substrate using the same treatment time. This indicates that the plasma etching is affected by the types of substrates, and flat substrates may be more beneficial for plasma etching. To clarify the morphology difference between thermal shrinking and plasma etching of PS nanospheres. The surface morphology of thermal-shrinking nanospheres was characterized (Figure S4). The conformal nanospheres on the bumpy Au electrodes and PET substrates were heated to 120 °C for 180 s. Different from the plasma-treated nanosphere array, the thermal-shrinking nanospheres agglomerated together, suggesting that plasma etching can better maintain the morphology of the nanospheres and avoid agglomeration.

### 3.2. Decoration of WS_2_ Sheets on the Conformal Nanospheres

**Figure 4:**
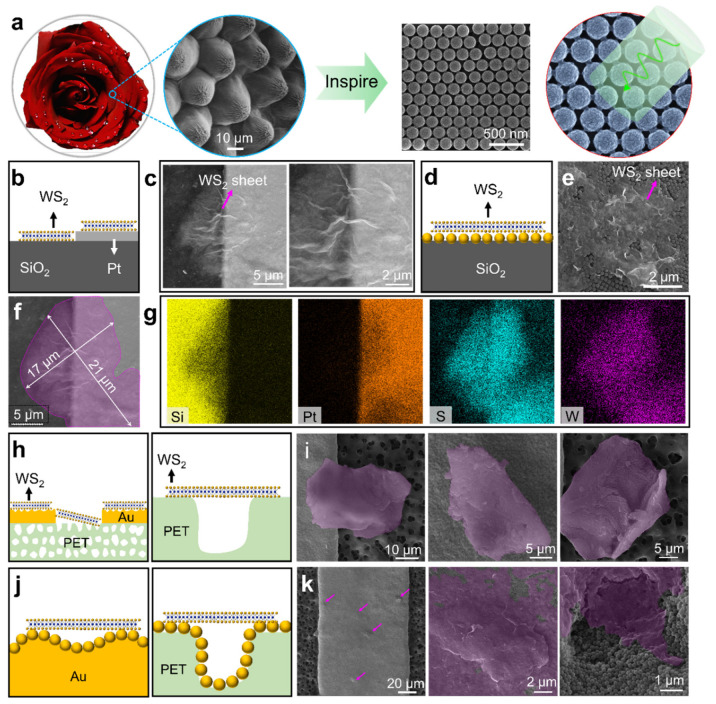
The fabrication of 2D WS_2_ sheets on rose-petal-inspired nanosphere array. (**a**) Biomimetic strategy for enhancing light harvesting using the nanosphere array; the photo and SEM image of rose petal were taken by the author. (**b**–**e**) Schematics and SEM images show WS_2_-decorated (**b**,**c**) flat substrates and (**d**,**e**) nanosphere-covered substrate. (**f**,**g**) SEM image and EDS mapping results of the WS_2_-decorated Pt and SiO_2_ substrates. (**h**–**k**) Schematics and SEM images show the WS_2_-decorated (**h**,**i**) Au and PET substrate and (**j**,**k**) conformal nanosphere-coated substrates. The WS_2_ sheets in the SEM images are highlighted with purple color.

Many studies have demonstrated that the rose petal is capable of enhancing light harvesting due to periodic hierarchical papillae arrays on its surface [57,58], and a number of artificial papillary micro/nanostructures have been fabricated for improving the light-capturing efficiency of solar cells [59], as well as surface-enhanced Raman scattering [60,61]. Inspired by this, 2D WS_2_ sheets were loaded on the nanosphere array to enhance the interaction of light and WS_2_ (Figure 4a). The WS_2_ sheets were deposited onto the flat and the curved substrates by immersing the plasma-treated substrates into WS_2_ suspension with a concentration of 1 mg/mL. After solvent evaporation, the WS_2_ sheets with a feature size of ~20 μm spontaneously buckled into nanowrinkles on the substrates (Figure 4b,c). For the nanosphere-covered substrates, the WS_2_ sheets wrap the nanospheres and form undulating films (Figure 4d,e). The energy-dispersive spectroscopy (EDS) mapping and spectra further demonstrate that the WS_2_ sheets were successfully loaded on the flat Pt and SiO_2_ substrate (Figure 4f,g), as well as the nanosphere-covered substrates (Figure S5). Different from the flat substrates, the WS_2_ sheets tend to agglomerate on the surface of the bumpy Au electrode and the porous PET substrate, spontaneously folding into crumples (Figure 4h,i). Besides, the crumpled WS_2_ sheets overlap on the electrode and the substrate. By contrast, the WS_2_ sheets uniformly spread onto the nanosphere-coated curved substrates, forming continuous films on the nanosphere array (Figure 4j,k). Impressively, plenty of WS_2_ sheets suspend over the nanosphere-coated microholes, leading to the formation of numerous WS_2_-capsuled micro/nanocavities. These results suggest that the conformally coated nanospheres on the curved substrates are beneficial in facilitating the attachment of 2D materials.

The absorbance spectra of the WS_2_ sheets on the flat SiO_2_ substrate and the nanosphere-covered flat SiO_2_ substrate were characterized to see the effect of hexagonally packed nanospheres on the light absorbance. Two feature exciton absorption peaks of WS_2_ induced by two direct bandgap transitions were observed in the spectra: A exciton peak (~500–550 nm) and B exciton peak (~600–650 nm) (Figure S6) [62,63]. For simplicity, the nanosphere is abbreviated as NS, and the WS_2_-decorated nanospheres are abbreviated as WS_2_-NS. The light absorption of the WS_2_ sheet was demonstrated to be enhanced by suspending the WS_2_ sheet onto the nanosphere array, and a more than 15% increase in the absorbance of A and B exciton peaks was observed. This result shows that the interaction between WS_2_ sheets and the incident light can be effectively improved by introducing sub-wavelength nanosphere arrays.

### 3.3. Light-Enhanced Room-Temperature Gas Sensing

**Figure 5:**
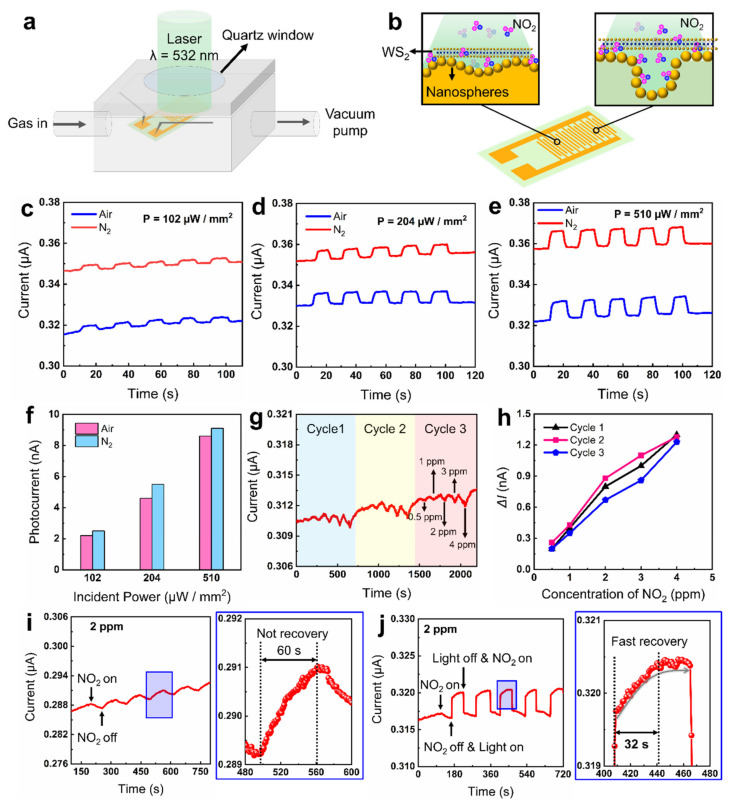
WS_2_-decorated nanospheres for light-enhanced room-temperature gas sensing. (**a**,**b**) Schematics of (**a**) the test platform and (**b**) the WS_2_-decorated nanospheres (WS_2_-NS) on the interdigital electrode for NO_2_ sensing. (**c**–**e**) Photoresponse of the WS_2_-NS sensor to air and N_2_ under various incident power conditions: (**c**) 102 µW/mm^2^, (**d**) 204 µW/mm^2^, (**e**) 510 µW/mm^2^. (**f**) Photocurrent of the sensor with the variation of the incident power. (**g**,**h**) Current changes with the variation of NO_2_ concentration from 0.5 to 4 ppm under room temperature (26 °C). (**i**) Cycling response of the gas sensor to 2 ppm NO_2_ gas. (**j**) Light-enhanced gas desorption. The incident power is 102 µW/mm^2^.

With the advantages of photoresponsive 2D WS_2_ sheets and 3D nanosphere-coated microarchitectures, light-enhanced pressure monitors and gas sensors with excellent performance may be developed. WS_2_-decorated nanospheres (WS_2_-NS) on bumpy Au electrodes and porous PET substrates were fabricated to investigate their performance in airtightness monitoring and gas sensing (Figure 5a,b). A gas chamber equipped with a quartz window and metal probes was used to characterize the performance of the sensor (Figure 5a; Figure S7). It is worth mentioning that an Au nanofilm was sputtered on the nanospheres to improve the conductivity. Firstly, the photocurrent of the WS_2_-NS sensor in dry air and N_2_ gas was characterized to see the effect of O_2_ on the photoelectric property of the sensor. By increasing the incident power from 102 to 510 μW/mm^2^, the photocurrent increased from 2.2 to 8.6 nA in air (Figure 5c–e). After replacing the air with N_2_, the photocurrent increased slightly (Figure 5f). The air used in this work was dry air, and the main difference in the components between the dry air and the N_2_ is that the air contains O_2_ molecules. Compared with pure N_2_, the O_2_ molecules absorbed from the air can act as electron acceptors to induce a slight decrease in the current. This result is consistent with that of previous work [52]. Huo et al. [52] also demonstrated that the absorbed O_2_ molecules act as electron acceptors that are able to accept electrons from WS_2_, inducing a decrease in overall conductivity. Since the O_2_-induced decrease in the photocurrent was very small (less than 1 nA), the dry air could be used as the purge gas for the developed gas sensor.

As a typical toxic gas, the detection of low-concentration NO_2_ gas is of great significance for early warning of hazardous chemical exposure [46]. For example, the detection of NO_2_ exposure in a chemical warehouse may provide an early warning of spontaneous combustion and explosion so as to reduce economic losses. However, most room-temperature NO_2_ sensors suffer from slow recovery speed, which limits their application in timely warning of hazardous exposure. To speed up the recovery time, light illumination may be a simple and fast method. Considering low dark current and porous microstructures, the NO_2_ sensing performance of the WS_2_-NS sensor was investigated. Firstly, the response of the sensor under various gas concentrations without light illumination was characterized (Figure 5g). The current decrease (ΔI) induced by the NO_2_ within 60 s was used to characterize the response of the gas sensor. As shown in Figure 5h, the current response increased from 0.2 to 1.3 nA with the increase in the NO_2_ concentration from 0.5 to 4 ppm. Besides, the gas sensor exhibited good reproducibility and linearity through three absorption–desorption cycles. Then, the response of the gas sensor to 0.1, 0.2 and 0.5 ppm NO_2_ gas was measured to characterize the detection limit of the gas sensor (Supplementary Materials, Figure S8a). As a result, the detection limit of the gas sensor was demonstrated to be 0.5 ppm. Moreover, the sensor exhibited good selectivity in the detection of NO_2_ gas (Figure S8b). When various target gases, including CH_2_O, NH_3_, CO and NO_2_, with a concentration of 1 ppm were assessed, the sensor only showed an obvious response to the exposure of NO_2_ gas. However, it is still hard to realize the total desorption of the absorbed NO_2_ gas at room temperature. To clarify whether the light illumination is beneficial for NO_2_ desorption, the current response of the sensor to 2 ppm NO_2_ gas under dark and light illumination was studied (Figure 5i,j). The result shows that total desorption of NO_2_ was difficult to realize within 60 s. Impressively, the recovery time decreases remarkably to 32 s after introducing 532 nm light illumination during the gas desorption. This demonstrates that the photoexcited carriers are capable of reducing the recovery time of the NO_2_ gas sensor. Fast recovery speed makes the developed room-temperature gas sensor a promising candidate for monitoring hazardous exposure.

### 3.4. Light-Enhanced Airtightness Monitoring

**Figure 6:**
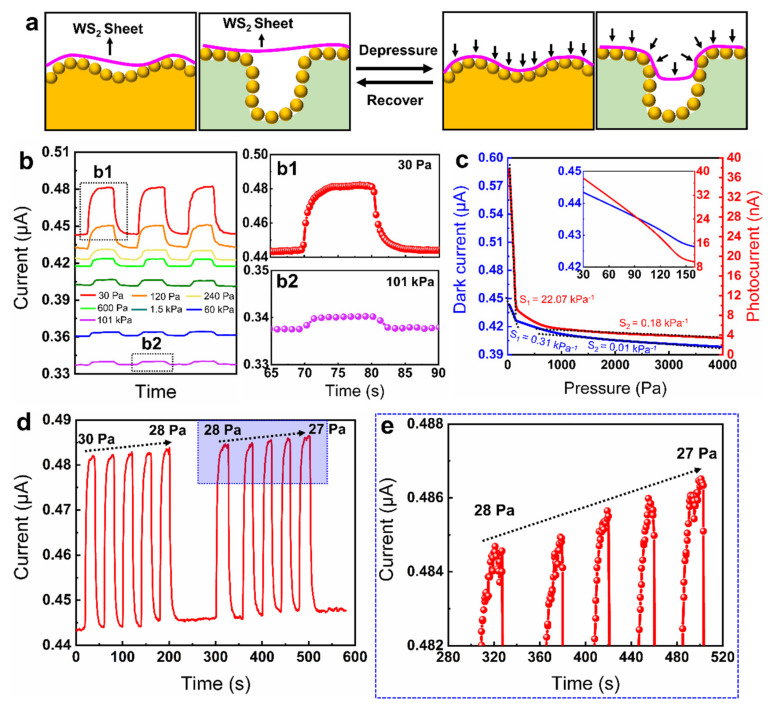
WS_2_-decorated nanospheres for photoresponsive pressure sensor. (**a**) Schematics illustrate the sensing mechanism of the WS_2_-NS pressure sensor. (**b**) Photoresponse of the WS_2_-NS sensor under various pressure conditions: (**b1**) 30 Pa; (**b2**) 101 kPa. (**c**) Dark current and photocurrent of the sensor with the variation of the pressure. (**d**) Photoresponse of the WS_2_-NS sensor a decrease in pressure from 30 to 27 Pa. (**e**) Enlarged view of the blue box in Figure 6d. The incident power is 102 µW/mm^2^.

The combination of 2D materials and conformal nanospheres on 3D microarchitectures provides a new avenue for designing gas sensors that are able to detect the gas pressure, which is crucial for gas detection under ultrahigh or ultralow ambient pressure. To address this challenge, photoenhanced pressure sensors based on the WS_2_-decorated nanospheres were designed (Figure 6a). For comparison, two types of sensors were fabricated: the WS_2_ sensor was obtained by directly depositing WS_2_ sheets on the interdigital electrode, and the WS_2_-NS sensor was achieved via coating the WS_2_ sheets onto the nanosphere-covered interdigital electrode. The photoresponse of the WS_2_-NS sensor under the pressure ranging from 0.03 to 101 kPa was characterized firstly (Figure 6b). Both the dark current and the photocurrent increased with the decrease in the air pressure (Figure 6c). For example, the photocurrent increased from 2.4 to 37.7 nA when the pressure was reduced from 101 to 0.03 kPa. Compared with the dark current, the photocurrent of the WS_2_-NS sensor is much more sensitive to pressure. The sensitivity (S) of the pressure sensor is defined as the ratio of the relative current change (ΔI/I_0_) to the pressure change (ΔP). A high sensitivity of 22.07 kPa^−1^ (~30–140 Pa) was demonstrated for the WS_2_-NS pressure sensor under light illumination, which is 71 times larger than that under dark conditions (~30–160 Pa). After that, the performance of the sensor responses to much lower pressure (e.g., 27 Pa) was characterized (Figure 6d). The results show that the sensor has excellent stability in the detection of pressure ranging from 0.027 to 101 kPa. Particularly, the sensitivity of the pressure sensor increased dramatically with the reduction in pressure below 200 Pa. For example, when the pressure is reduced from 30 to 27 Pa, the dark current and the photocurrent of the sensor increased by 4.1 and 1.3 nA, respectively (Figure S9). Currently, the lower and upper application limits of the WS_2_-NS sensor are 27 Pa and 101 kPa, respectively. In the future, we will continue to improve the test conditions and explore the performance of the sensor at much lower pressures (e.g., 10^−4^ Pa).

**Figure 7:**
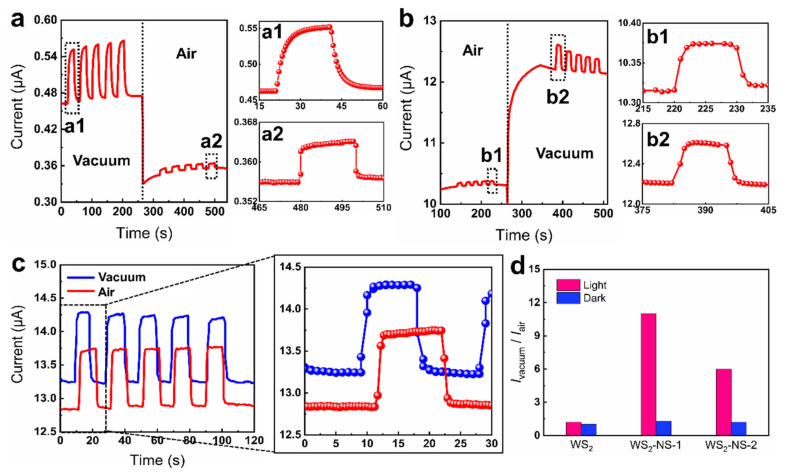
Demonstration of the WS_2_-NS pressure sensor for airtightness monitoring. (**a**,**b**) Controlling the photoresponse of the WS_2_-NS sensor under vacuum and air conditions by varying the concentration of WS_2_ suspension: (**a**) 1 mg/mL; (**b**) 2 mg/mL. The obtained sensors are named WS_2_-NS-1 and WS_2_-NS-2, respectively. (**c**) Dynamic response of the WS_2_ sensor without nanosphere coatings. The incident power is 510 µW/mm^2^. (**d**) The ratio of the dark/photocurrent in vacuum to that in air.

Because of high sensitivity to low pressure and fast response speed, the fabricated WS_2_-NS pressure sensor is suitable for airtightness monitoring. Two pressure sensors named WS_2_-NS-1 and WS_2_-NS-2 were fabricated by coating 1 and 2 mg/mL WS_2_ suspension onto the nanosphere-covered interdigital electrodes, so as to explore their performance in airtightness monitoring. Surprisingly, the photocurrent of the WS_2_/NS-1 sensor under vacuum (120 Pa) was 91 nA, 11 times higher than that in air (101 kPa) (Figure 7a). A fast and sharp decrease in the current was observed when the vacuum was broken by opening the air inlet. Accordingly, the photocurrent also decreased dramatically with the increase in pressure. This makes the WS_2_/NS sensor a promising candidate for monitoring airtightness in situations where a vacuum is needed. Compared with the WS_2_-NS-1 sensor, the WS_2_-NS-2 sensor exhibited a much higher dark current and photocurrent under both vacuum and air conditions (Figure 7b). Similarly, the WS_2_-NS-2 sensor was also demonstrated to be able to detect gas leakage in a dynamic-pressure environment.

By contrast, the current of the WS_2_ sensor without conformal nanospheres remained stable under both vacuum and air conditions (Figure 7c). The dark current of the sensor in air and under vacuum was as large as 12.8 and 13.2 μA, respectively, due to direct contact between the WS_2_ sheets and the electrode. Correspondingly, the photocurrents of the WS_2_ sensor were 0.87 μA in air and 1.04 μA under vacuum, respectively. The performance of the pressure sensor in airtightness monitoring was evaluated by the ratio of dark/photocurrent under vacuum (I_vacuum_) to that in air (I_air_). As shown in Figure 7d, the ratio of dark current under vacuum to that in air was lower than 1.5. Except for the WS_2_ sensor, both WS_2_-NS-1 and WS_2_-NS-2 sensors showed a vacuum-to-air photocurrent ratio higher than 6. This result further demonstrates the significance of conformal nanospheres in enhancing the sensitivity of the pressure sensor. For WS_2_-decorated conformal nanospheres on undulating substrates, numerous micro/nano air-pockets can be induced, and the contact between WS_2_ sheets and the nanospheres is poor in air. The contact area between the WS_2_ sheets and the nanospheres can be improved dramatically by depressuring, so as to induce a sharp decrease in the resistance. Similarly, depressure-induced better ohmic contact between the WS_2_ sheets and the substrates is also beneficial for the separation of electron–hole pairs, resulting in a much higher photocurrent. The combination of 2D materials and conformal nanospheres on curved surfaces provides a new avenue for the detection of airtightness, which may find applications in monitoring the airtightness of space aircraft.

## 4. Conclusions

In summary, a highly efficient self-assembly method was developed for the fabrication of conformal nanospheres on microarchitectures such as metal microbumps and polymer microholes. Plasma treatment plays a significant role in enhancing the hydrophilicity of the target substrates, and it is crucial for the conformal assembly of nanospheres. Different from previous methods, this approach is much more universal for fabricating conformal nanostructures on microarchitectures with various surface chemicals. Besides, plasma treatment was also demonstrated to be able to regulate the feature size of the conformal nanospheres. Similar to the hierarchical papillae array on the rose petal, the conformal nanospheres with nanogaps were demonstrated to be able to enhance the light harvesting of WS_2_ sheets. Based on this, a light-enhanced airtightness monitor and a room-temperature gas sensor with a fast recovery speed (<35 s) and low detecting limit (500 ppb) were developed. This work provides a universal method for fabricating conformal nanospheres on arbitrary substrates, especially for curved surfaces, which is promising for designing and fabricating artificial metasurfaces, biomimetic microarchitectures and other functional structures.

## Data Availability

The data presented in this study are available on request from the corresponding author.

## Supplementary Materials

The following are available online at https://www.mdpi.com/article/10.3390/nano11071829/s1, Figure S1: Controlling the feature size and the arrangement of the nanospheres on flat substrate by varying the plasma treatment time, Figure S2: Schematics illustrate the assembly mechanism of conformal nanospheres on flat and curved substrates, Figure S3: Controlling the feature size of the nanospheres on curved substrate by varying the plasma treatment time, Figure S4: Surface morphology of the PS nanospheres on curved substrates after thermal treatment, Figure S5: EDS analysis of the WS2 sheets loaded on the nanosphere array, Figure S6: Absorbance spectra of the WS2 sheets. Figure S7: Photographs show the mass flow controllers and the test chamber. Figure S8: Detection limit and selectivity of the gas sensor. Figure S9: Dark current and photocurrent of the sensor with the variation of the pressure.
