# Supplementary material for: Conformal Self-Assembly of Nanospheres for Light-Enhanced Airtightness Monitoring and Room-Temperature Gas Sensing
Source: Nanomaterials (Basel). 2021 Jul 14;11(7):1829. doi: 10.3390/nano11071829 (PMC8308308; doi:10.3390/nano11071829)
Supplement: Supplementary file 1 [file nanomaterials-11-01829-s001.zip › nanomaterials-1289078-supplementary.pdf]

Supplementary Materials

Article

Conformal Self-Assembly of Nanospheres for Light-Enhanced Airtightness Monitoring and Room-Temperature Gas Sensing

Qirui Liu [1], Yinlong Tan [2,*], Renyan Zhang [1], Yan Kang [1], Ganying Zeng [1], Xiaoming Zhao [3] and Tian Jiang [2,*]

[1] College of Advanced Interdisciplinary Studies, National University of Defense Technology,
Changsha 410073, China; lqrkili@163.com (Q.L.); ryancms@sina.cn (R.Z.); 13321112309@163.com (Y.K.); zengganying@nudt.edu.cn (G.Z.)

[2] Beijing Institute for Advanced Study, National University of Defense Technology, Beijing 100000, China

[3] State Key Laboratory of High Performance Computing, College of Computer Science and Technology,
National University of Defense Technology, 410073 Changsha, China; zxm_0911@126.com

* Correspondence: tanyinlong15@nudt.edu.cn (Y.T.); tjiang@nudt.edu.cn (T.J.)

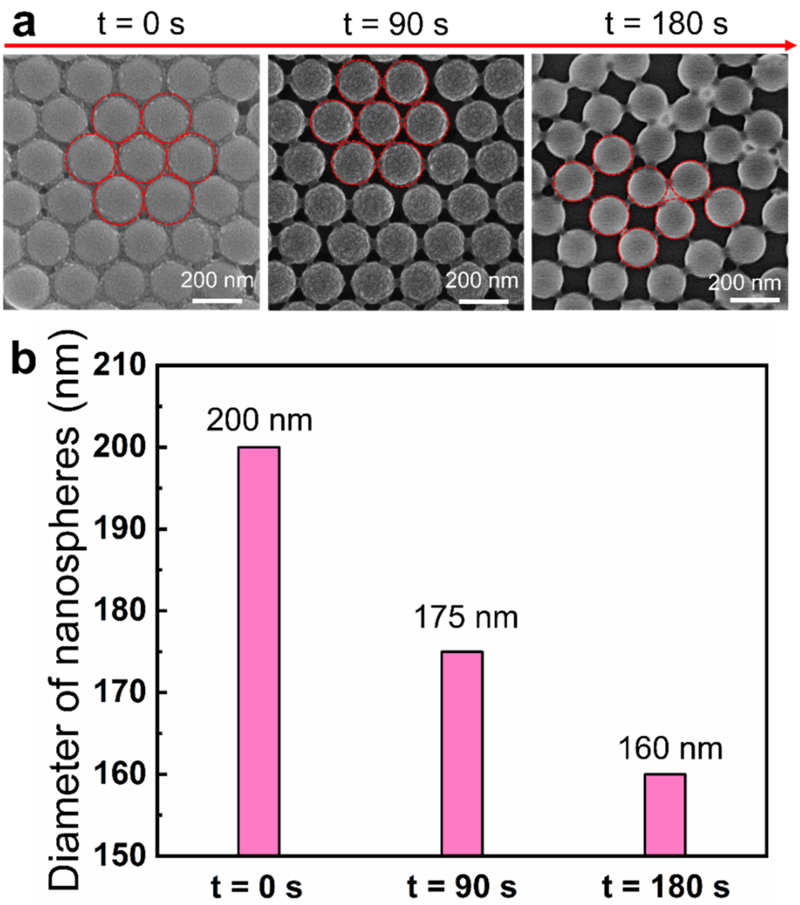

Figure S1. Controlling the feature size and the arrangement of the nanospheres on flat substrate by varying the plasma treatment time. (**a**) SEM images show the topography of the self-assembly PS nanospheres on the substrates with variation of the plasma treatment time. (**b**) The diameter of the nanospheres changes with the variation of plasma treatment time.

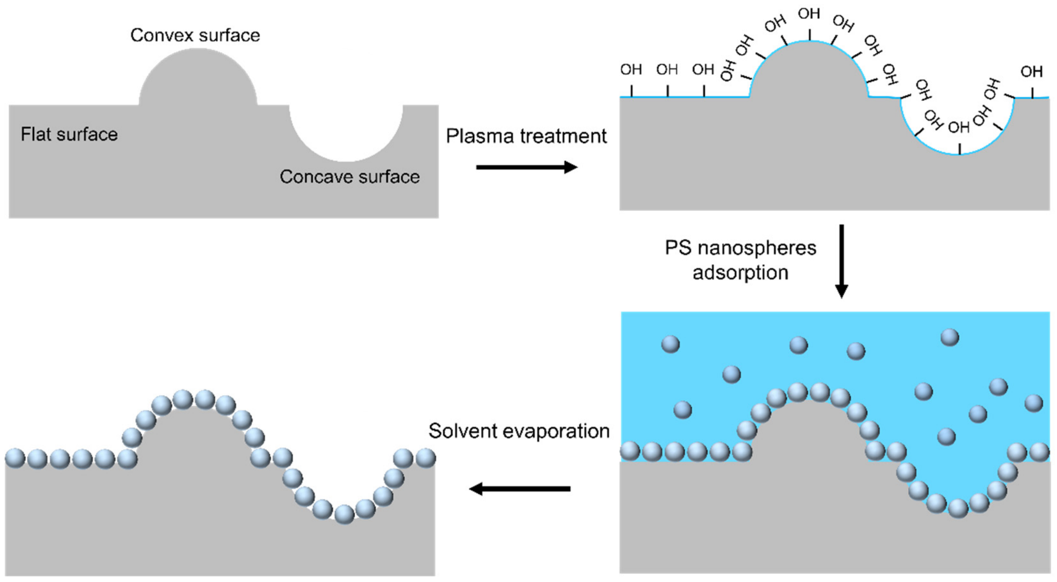

Figure S2. Schematics illustrate the assembly mechanism of conformal nanospheres on flat and curved substrates. After plasma treatment, numerous hydroxyl groups are introduced onto the substrates and the substrates become superhydrophilic. The PS nanospheres spontaneously adhere to the substrates and then conformal nanospheres coating can be induced after solvent evaporation.

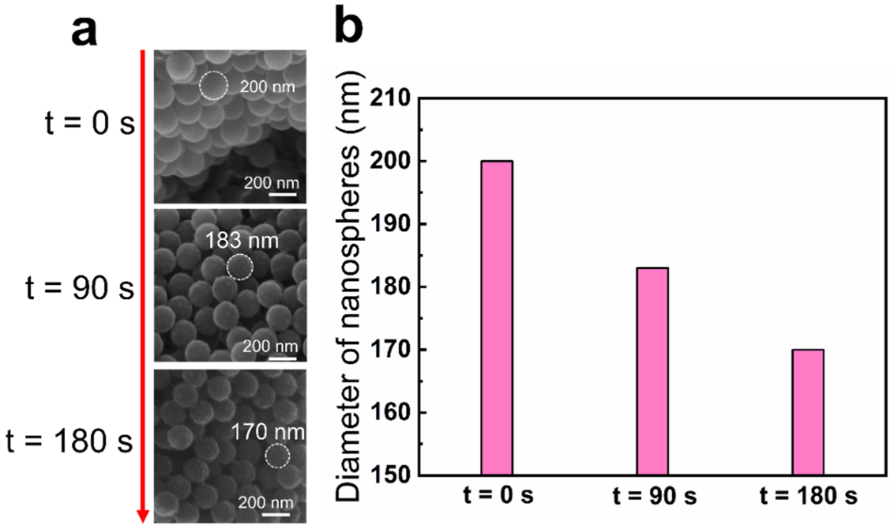

Figure S3. Controlling the feature size of the nanospheres on curved substrate by varying the plasma treatment time. (**a**) SEM images show the topography of the self-assembly nanospheres on the substrates with variation of the plasma treatment time. (**b**) The diameter of the nanospheres changes with the variation of plasma treatment time.

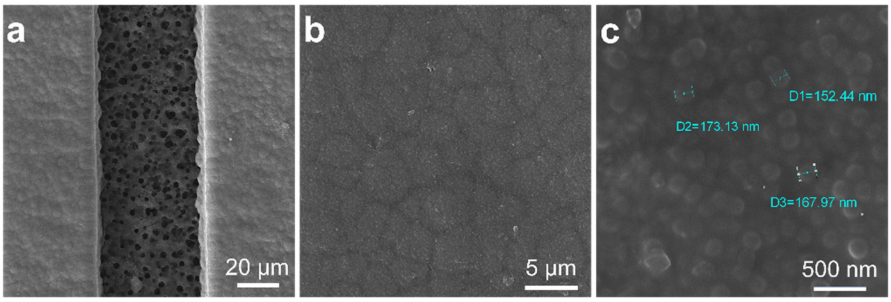

Figure S4. Surface morphology of the PS nanospheres on curved substrates after thermal treatment.

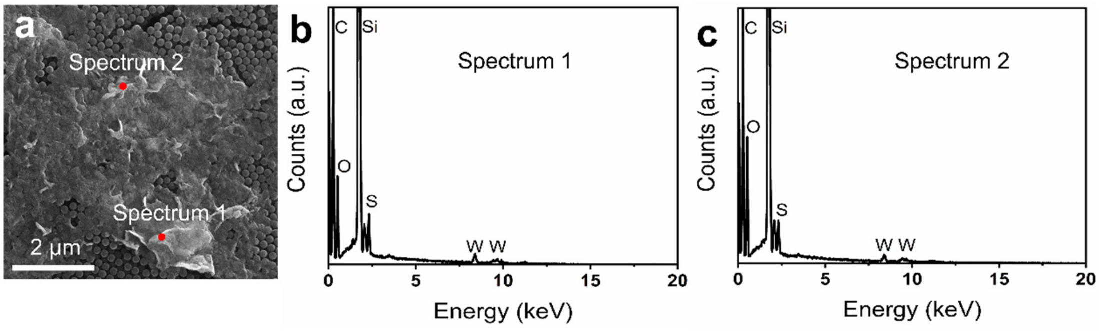

Figure S5. EDS analysis of the WS_2 sheets loaded on the nanospheres array. (**a**) The SEM image of the WS_2 sheets on the nanospheres. (**b**,**c**) EDS results of two points selected on the crumpled WS_2 sheets.

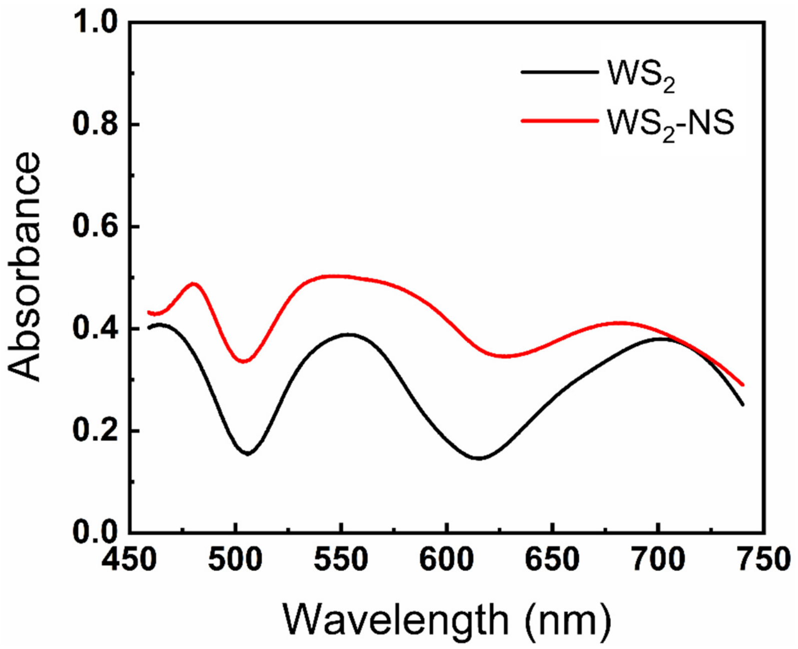

Figure S6. Absorbance spectra of the WS_2 sheets on the flat SiO_2 substrate (WS_2, black line) and the nanospheres-covered flat SiO_2 substrate (WS_2-NS, red line).

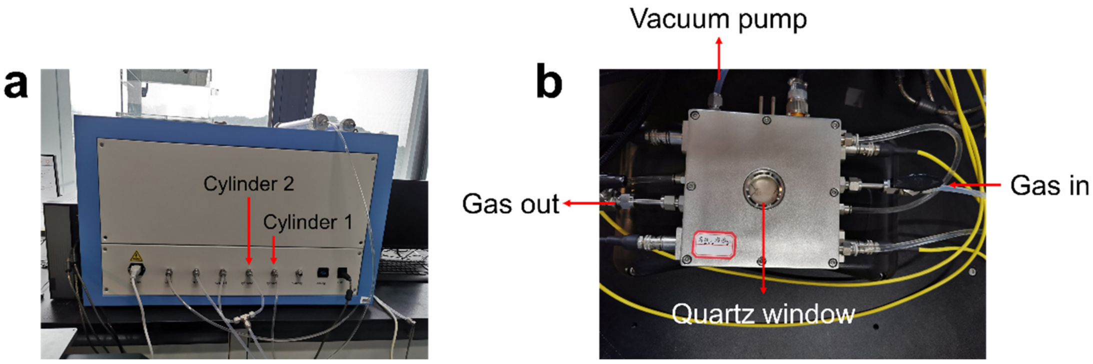

Figure S7. Photographs show **a**) the mass flow controllers and **b**) the test chamber.

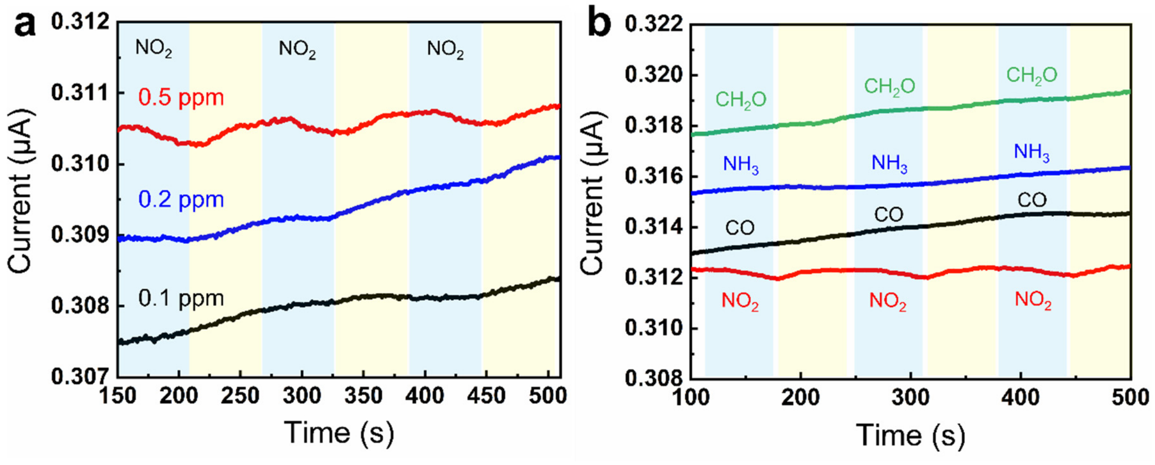

Figure S8. Detection limit and selectivity of the gas sensor. (**a**) The response of the gas sensor to various concentration (0.1, 0.2, and 0.5 ppm) of NO_2 gas. (**b**) The response of the gas sensor to various kinds of target gas including CH_2O, NH_3, CO and NO_2. The concentration of the target gases is 1 ppm.

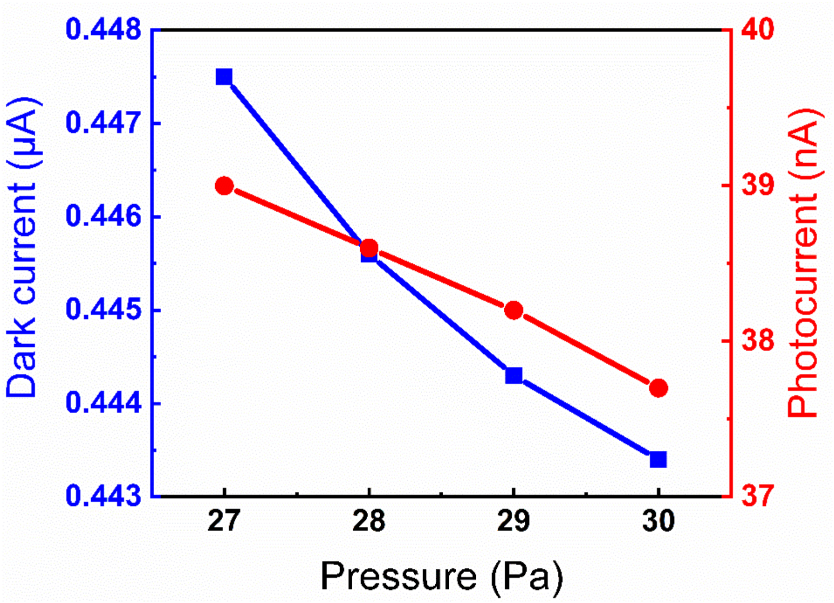

Figure S9. Dark and photocurrent of the sensor with the variation of the pressure ranging from 27 to 30 Pa.
